# Hormonal Therapy for Gynecological Cancers: How Far Has Science Progressed toward Clinical Applications?

**DOI:** 10.3390/cancers14030759

**Published:** 2022-02-01

**Authors:** Saikat Mitra, Mashia Subha Lami, Avoy Ghosh, Rajib Das, Trina Ekawati Tallei, Fatimawali, Fahadul Islam, Kuldeep Dhama, M. Yasmin Begum, Afaf Aldahish, Kumarappan Chidambaram, Talha Bin Emran

**Affiliations:** 1 Department of Pharmacy, Faculty of Pharmacy, University of Dhaka, Dhaka 1000, Bangladesh; saikat-2018926336@pharmacy.du.ac.bd (S.M.); mashiasubha-2018526349@pharmacy.du.ac.bd (M.S.L.); avoy-2018626384@pharmacy.du.ac.bd (A.G.); rajib-2016714522@pharmacy.du.ac.bd (R.D.); 2 Department of Biology, Faculty of Mathematics and Natural Sciences, Sam Ratulangi University, Manado 95115, Indonesia; trina_tallei@unsrat.ac.id; 3 The University Center of Excellence for Biotechnology and Conservation of Wallacea, Institute for Research and Community Services, Sam Ratulangi University, Manado 95115, Indonesia; fatimawali@unsrat.ac.id; 4 Pharmacy Study Program, Faculty of Mathematics and Natural Sciences, Sam Ratulangi University, Manado 95115, Indonesia; 5 Department of Pharmacy, Faculty of Allied Health of Sciences, Daffodil International University, Dhaka 1207, Bangladesh; fahadul29-774@diu.edu.bd; 6 Division of Pathology, ICAR-Indian Veterinary Research Institute, Izatnagar, Bareilly 243122, Uttar Pradesh, India; kdhama@rediffmail.com; 7 Department of Pharmaceutics, College of Pharmacy, King Khalid University, Abha 61441, Saudi Arabia; ybajen@kku.edu.sa; 8 Department of Pharmacology and Toxicology, College of Pharmacy, King Khalid University, Abha 62529, Saudi Arabia; adahesh@kku.edu.sa (A.A.); kumarappan@kku.edu.sa (K.C.); 9 Department of Pharmacy, BGC Trust University Bangladesh, Chittagong 4381, Bangladesh

**Keywords:** hormonal therapy, gynecological cancers, aromatase inhibitors, anti-estrogen, GnRH agonist

## Abstract

**Simple Summary:**

The most common therapies for severe and recurrent gynecological cancers are hormone therapy and chemotherapy, and responsiveness to therapy is a key component in prognosis and survivability. Hormone therapy has recently been demonstrated to be an excellent cancer treatment approach. Hormone treatment for gynecological cancers is taking drugs that decrease hormone levels or impede their biological activity, halting or slowing cancer progression. Hormone therapy works by suppressing the multiplication of cancer cells triggered by hormones. Hormonal therapy, such as progestogens or tamoxifen, is frequently recommended for patients with hormone-sensitive recurrent or metastatic gynecological cancers, but response rates and therapeutic effects are inconsistent. Therefore, we discuss the pathogenesis of gynecological malignancies from the hormonal landscape and the use of hormonal therapies toward clinical applications.

**Abstract:**

In recent years, hormone therapy has been shown to be a remarkable treatment option for cancer. Hormone treatment for gynecological cancers involves the use of medications that reduce the level of hormones or inhibit their biological activity, thereby stopping or slowing cancer growth. Hormone treatment works by preventing hormones from causing cancer cells to multiply. Aromatase inhibitors, anti-estrogens, progestin, estrogen receptor (ER) antagonists, GnRH agonists, and progestogen are effectively used as therapeutics for vulvar cancer, cervical cancer, vaginal cancer, uterine cancer, and ovarian cancer. Hormone replacement therapy has a high success rate. In particular, progestogen and estrogen replacement are associated with a decreased incidence of gynecological cancers in women infected with human papillomavirus (HPV). The activation of estrogen via the transcriptional functionality of ERα may either be promoted or decreased by gene products of HPV. Hormonal treatment is frequently administered to patients with hormone-sensitive recurring or metastatic gynecologic malignancies, although response rates and therapeutic outcomes are inconsistent. Therefore, this review outlines the use of hormonal therapy for gynecological cancers and identifies the current knowledge gaps.

## 1. Introduction

Cancer is a leading cause of death worldwide. Gynecological cancers are the most prevalent cancer in women. Cervical cancer is the most common type of gynecological cancer [1]. Gynecological cancer refers to any cancer that develops in a woman’s reproductive organs. Gynecological cancers can start anywhere in a woman’s pelvis, the area beneath her stomach, and between her hip bones [2]. Cervical cancer, vulvar cancer, vaginal cancer, uterine cancer, and ovarian cancer are the five categories of gynecological cancer. The cervix, the lower and narrow end of uterus, is where cervical cancer develops [3]. The developing world bears a disproportionately high burden of cervical cancer, accounting for 85% of the approximately 493,000 new cases and 273,000 deaths annually. Cervical cancer is an issue in areas where most people are underprivileged and women’s socioeconomic position is low, and ethnicity can also be a risk factor [4]. Cervical cancer is the fourth most common cancer in women and the fourth leading cause of cancer deaths. Premalignant cervical intraepithelial neoplasia (CIN) precedes cervical cancer. CINs (CIN1, CIN2, CIN3) can be efficiently cured by uncomplicated surgery, but they carry a risk of future pregnancy problems [5]. Ovarian cancer is the most common cause of mortality from gynecological cancer. Around 70% of patients who have ovarian cancer are diagnosed at stage III/IV and 75% of these cases deteriorate within two years of first-line therapy, making it unlikely that they will be cured. Though salvage chemotherapy for recurrent cancer can produce objective tumor responses, it does not always result in longer progression-free or overall survival [6,7]. Ovarian cancer is believed to be influenced by the pituitary gonadotropins follicle-stimulating hormone (FSH) and luteinizing hormone (LH), and by progesterone, androgens, IGF-I, and estrogens [8].

Uterine cancer, also known as endometrial cancer, starts in the uterus, in the layer of cells that make up the uterus lining (endometrium) [9]. Endometrial cancer is the sixth most prevalent cancer in women and the fifteenth most common cancer in the general population. In 2018, there were around 380,000 new cases [10]. Vaginal cancer has five subtypes: sarcomas, squamous cell carcinoma, clear cell adenocarcinoma, adenocarcinoma, and melanoma [11]. The 5-year survival rate for women with vaginal cancer is 49%. If the cancer is diagnosed early, prior to spreading outside of the vaginal wall, the 5-year survival rate is 66% [12]. Another type of gynecological cancer is vulvar cancer, which begins in the vulva. Patients with vulvar cancer have a 5-year survival rate of around 70% [13].

Hormone therapy and chemotherapy are the most common treatments for severe and recurrent gynecological malignancies, and responsiveness to therapy is a critical factor affecting prognosis and survival. Hormone therapy has become more frequently used in recent years as a result of the harsh side effects of chemotherapy. Hormone treatment for cancer involves taking medications that inhibit the biological activity or reduce the level of hormones in order to prevent or reduce cancer growth. Hormone therapy either inhibits the production of hormones or stops hormones from causing cancer cells to multiply and divide. However, it is not effective against all cancers [14]. The treatment of patients with gynecological cancers has advanced significantly in recent years [15]. 

The goal of this study was to convey the current state of gynecological cancer research, with a focus on hormone therapy. Extensive literature searches and reviews were conducted in order to determine the pathogenesis and possible therapy of hormones. The aim of this review is to understand the use of hormones in the treatment of gynecological cancers.

## 2. Pathogenesis of Gynecological Cancers: Hormonal Landscape

### 2.1. Cervical Cancer

**Figure 1:**
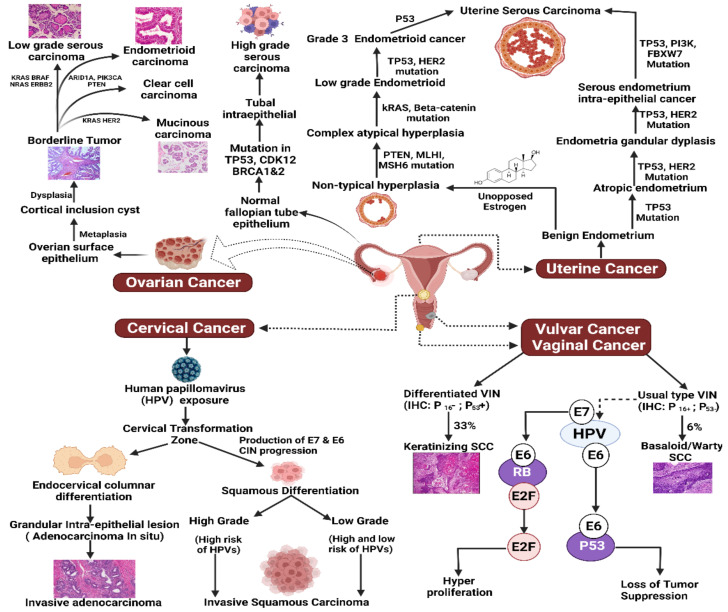
Pathogenesis of gynecological cancers. Ovarian: Borderline tumors can form from the ovarian surface epithelium and can develop into low grade serous, endometrioid, clear cell, and mucinous carcinoma by mutation and alteration of certain genes, e.g., KRAS, BRAF, NRAS, HER2, CTNNB1, BRAF, ERBB2, ARID1A, PIK3CA, PTEN, HER2, etc. High-grade serous carcinoma is developed from normal fallopian tube epithelium by the mutation of TP53, CDK12, BRCA1 and 2. Uterine: Uterine cancer result from TP53, HER2, PI3K, FBXW7, KRAS, PTEN, MLHI, MSH6, Beta- catenin mutation, and CTNNBI alteration. Vulvar: (uVIN: E6 degrades the tumor suppressor p53; E7 inactivates the tumor suppressor RB and releases E2F resulting in hyperproliferation.) (dVIN: Chronic dermatoses, especially Lichen sclerosus and Lichen planus, can progress to dVIN and SCC), (VIN, vulvar intraepithelial neoplasia). Vaginal: TP53 gene alteration and HPV 16 and 18, etc., are the importance carcinogenic factors for both HPV-dependent or -independent vaginal cancer. Cervical: Production of E7 and E6, infection with HPV, CIN progression cause differentiation in squamous cervical cells and cause invasive adenocarcinoma.

Chronic infection with oncogenic HPV strains is the most important etiological factor in the development of cervical cancer. The production of two viral oncoproteins, E7 and E6, in parabasal or basal cells, can trigger and maintain neoplastic development [16]. In pre-invasive squamous cell carcinoma of the cervix, defective cells are restricted to the epithelium. Cervical intraepithelial neoplasia (CIN) is a noninvasive disorder that is linked to HPV integration and infection. CIN progresses from early modifications involving the deeper layers of the epithelium (CIN1) to later stages involving the entire epithelium (CIN3), which equates to squamous cell carcinoma (Figure 1) [17]. Cervical adenocarcinoma is a malignant neoplasm involving the cervical glandular epithelium. Adenocarcinoma exists in a variety of forms; the majority of these have identical etiology and risk factors to squamous cell carcinoma. Seventy percent of adenocarcinomas are endocervical-type mucinous adenocarcinomas [18,19].

Squamous cell carcinoma is the more prevalent type of cervical cancer. The most prevalent cause of cervical cancer, HPV, has a glucocorticoid/progesterone response element upstream of the common E7/E6 promoter, and progesterone increases the viral DNA’s capacity to transform cells [20,21,22]. Papillomavirus lesions are aggravated through pregnancy when progesterone levels are high, and oral contraceptives containing progestogen are a risk factor for cervical cancer development in HPV-positive individuals. Estrogen activates human HPV development by upregulating progesterone receptors [22,23]. Although the relationship between estradiol (E2) replacement or selective ER modulator (SERM) treatment and cervical cancer is contentious [24], the E2-ERα and progesterone–progesterone receptor (P4-PR) signaling pathways may be implicated in the progression and/or development of cervical cancer. PR is a ligand-dependent therapeutic target for cervical cancer, much as it is for endometrial cancer. While PR was produced in 100% of cervical malignancies in a mouse model, it is produced in only 20–40% of human cervical malignancies [25,26]. Estrogen promotes the growth of E6 and E7 oncogenes, which are thought to be the primary causes of cervical cancer. Furthermore, ERα and estrogen are essential for cervical carcinogenesis, while SERMs suppress cervical cancer in HPV-associated cervical cancer mouse models [27]. Estrogens stimulate cervical carcinogenesis, while progesterone prevents it, according to data from HPV transgenic mouse models [27,28].

### 2.2. Ovarian Cancer

The World Health Organization (WHO) histological classification of ovarian cancers is based on histogenetic principles, and this classification categorizes ovarian tumors based on their derivation from coelomic surface epithelial cells, germ cells, and mesenchyme (the stroma and the sex cord). The bulk of malignant ovarian tumors are epithelial ovarian tumors, which are further classified into histological types as follows: carcinosarcoma, mixed epithelial tumor, undifferentiated carcinoma, and others are examples of serous, mucinous, endometrioid, clear cell, transitional cell cancers (Brenner tumors), and others [29]. The most prevalent subtype of ovarian cancer is ovarian serous carcinoma. It manifests as either low-grade (10% of all serous subtype tumors) or high-grade carcinoma (90% of all the serous subtype tumors) [30]. Non-epithelial ovarian cancers are a rare but intriguing type of malignancies that can be particularly difficult to treat due to their non-epithelial nature. These tumors account for just 10–15% of all ovarian malignancies when taken as a whole, and they may arise at any age, from infancy to old age. As a general phrase, this covers a variety of tumors originating from germ cells, sex cord stromal cells, and other forms of ovarian cancer that are exceedingly uncommon, including small-cell carcinomas and sarcomas [31]. Conversely, epithelial ovarian tumors can be type I or type II. Examples of type I epithelial tumors, which grow slowly, are clear cell carcinomas, endometrioid, and low-grade micropapillary serous carcinomas. Examples of type II epithelial tumors include high-grade serous carcinoma, undifferentiated carcinoma, and carcinosarcoma. Borderline tumors begin as ovarian surface epithelium and progress to low-grade serous, endometrioid, clear cell, and mucinous carcinomas by mutation and change of certain genes such as KRAS, BRAF, NRAS, HER2, CTNNB1, BRAF, ERBB2, ARID1A, PIK3CA, PTEN, HER2, and others. In high-grade serous tumors, high-grade serous cancers that arise from normal fallopian tube epithelium by mutations in TP53 or BRCA1, BRCA2, MLH1, or MSH2 are thought to be the cause. [32]. Instead of traditional metastasis through the hematogenous pathway, ovarian cancers metastasize through the peritoneal circulation, which is involved in the formation of ascites [33].

CA-125 is the most well-known ovarian cancer biomarker. It is a high-molecular-weight transmembrane glycoprotein that is expressed by coelomic- and Müllerian-derived epithelia, such as those in the fallopian tube, endometrium, and endocervix [34]. Gene expression in ovarian cancer shows both how the cancer looks and how it behaves. Clear cell ovarian cancer is different from other cancers that have a poor prognosis because of this [35]. The most important thing about any tumor is the combination of genetic changes that make it grow and make it spread. In this area, ovarian tumors show a lot of different types. These types of tumors usually have changes in a lot of different genes, such as KRAS, BRAF, PTEN, TFG-R, and β–catenin [36]. Following the implantation of ovarian cancer cells, the resulting inflammation causes the release of cytokines such as interleukin (IL)-1, -8, and -6 by peritoneal cells and their associated stromal and immune cells, which drive tumor angiogenesis and ascites formation by enhancing tumor cell VEGF secretion [37]. In spite of their variations, each of these ovarian cancer metastatic processes are essentially driven by cell migration, involving cycles of cell adhesion, actin polymerization, and actomyosin contraction [38]. Furthermore, ovarian cancer cells undergo epithelial–mesenchymal transition driven by ROCK/Rho signaling (Figure 1) [37].

Microfibrillar-associated protein 5 stimulates ovarian cancer motility and metastatic potential via the Ca^2+^-dependent focal adhesion kinase/cAMP response element-binding protein/troponin C type 1 signaling pathway [39]. Versican is a major upregulated gene in CAFs that enhances ovarian cancer cell motility and invasion by activating NF-κB and upregulating the expression of MMP-9, CD44, and hyaluronan-mediated motility receptors in cancer cells [40,41]. The c-myc oncogene and HER-2/neu are amplified and overexpressed in 20–30% of epithelial ovarian cancers [42].

Hormone treatment employs the use of prescription medications to prevent or reduce the action of hormones such as estrogen. This is significant because certain types of ovarian cancer cells require those hormones to thrive and spread in the body [43]. Exaggerated stimulation of ovarian tissues by the pituitary gonadotropins follicle-stimulating hormone (FSH) and luteinizing hormone (LH) leads to ovarian cancer [44]. The longer a woman is subjected to estrogen, the greater her vulnerability to ovarian cancer is predicted to be. Because large amounts of estrogen are only present throughout a woman’s reproductive years, the longer she menstruates, the greater her risk. Childbearing can minimize risk by giving a woman nine-month “rests” from ovulation during pregnancy, lowering her total estrogen exposure. When taken for more than three cycles, the medications clomiphene citrate and pergonal, which are routinely used to treat infertility, appear to raise the risk of ovarian cancer [45].

### 2.3. Uterine and Endometrial Cancer

Uterine sarcoma, uterine carcinosarcoma, uterine clear cell carcinoma, and uterine papillary serous carcinoma are the most prevalent uterine and endometrial cancers. Uterine cancer has four stages: stage I, in which the cancer is confined to the uterus, stage 2, in which it has progressed to the cervix, stage III, in which the cancer has migrated to the vaginal canal, the lymph nodes, or the ovaries, and stage IV, in which the cancer has spread to the rectum or bladder, or organs other than the uterus, such as the lungs or bones [46].

Endometrial cancer develops when normal endometrial cell development is disrupted. Endometrial hyperplasia (overgrowth of endometrial cells) is a risk for adenocarcinoma since hyperplasia can and often does progress to adenocarcinoma; however, cancer can occur in the absence of hyperplasia [47]. This arises from endometrial hyperplasia brought on by uncontrolled estrogenic stimulation, and involves the production of progesterone and estrogen receptors by the estrogen passageway. There are two types of endometrial carcinoma, estrogen-dependent (type I) or estrogen-independent (type II). Clinical studies indicate that type I carcinomas are associated with low risk of lymph node metastasis, favorable prognosis, early cancer stage at diagnosis, and minimal myometrial invasion, whereas type II carcinomas are associated with high risk of lymph node metastasis, poor prognosis, advanced cancer stage at diagnosis, and deep myometrial invasion (Figure 1) [48].

The cancer usually progresses to the serosa and myometrium initially, before progressing to other pelvic and reproductive structures. Whenever the lymphatic system is involved, the para-aortic and pelvic lymph nodes are typically the first to be affected, however, unlike in cervical cancer, there is no specific pattern [49]. Endometrial cancer development is influenced by genes of grade 3. Mutations in a tumor suppressor gene, usually *PTEN* or *TP53*, are common. *PTEN* has a loss-of-function or null mutation in 50% of endometrioid malignancies and 20% of endometrial hyperplasias, making the PTEN protein either less efficient or completely inactive [50]. The mTOR/PI3K pathway is upregulated when *PTEN* function is lost, which drives cell proliferation. In endometrial cancers, the *TP53* pathway can either be repressed or highly active. Overexpression of a mutant form of *TP53* causes endometrial cancers to become more aggressive (Figure 1) [51].

Loss of function mutations to p27 and PTEN are linked to a favorable prognosis. In 20% of serous and endometrioid carcinomas, the oncogene Her2/neu is amplified or overexpressed. Mutations in CTNNB1 (encoding the β-catenin protein) are identified in 14–44% of endometrial malignancies and may suggest a favorable prognosis. In almost all endometrial cancers involving squamous cells, CTNNB1 alterations are present [51]. Although FGFR2 alterations are present in about 10% of endometrial malignancies, their impact on prognosis is unknown [50]. *SPOP* is another tumor suppressor gene that was reported to be mutated in 8% of serous endometrial carcinomas and 9% of clear cell endometrial carcinomas [52].

Distinct mutations are associated with type I and II malignancies. *ARID1A*, which often carries a point mutation in type I endometrial cancer, is also mutated in approximately 26% of endometrial clear cell carcinomas and 18% of serous carcinomas [53]. In both type I and II malignancies, *PIK3CA* is frequently mutated [51]. *KRAS* is also often mutated [54].

Gonadotropin-releasing hormone analog (GnRHa) was able to inhibit ovarian cancer cell growth by increasing inositol phosphate levels and initiating protein kinase pathways such as ERK1/2, and also to stimulate ovarian cancer cell apoptosis by increasing expression of apoptosis-associated genes and stimulating the Fas system [55]. Inadequate progesterone leads to unregulated estrogen activity, which can lead to adenocarcinoma and endometrial hyperplasia [56]. 

### 2.4. Vaginal Cancer

Vaginal cancer is a clinically diverse disease. Although HPV is a common cause of vaginal tumors, there are also carcinogenic mechanisms which are HPV-independent [57]. There are varying stages of histologic differentiation in vaginal cancer: invasive cancer, potential microinvasive carcinoma, carcinoma in situ, and vaginal intraepithelial neoplasia (VAIN) (from least to most malignant) [18,58]. *TP53* gene alterations are the principal carcinogenic factor in vulvar cancer and HPV 18 and 16 have a prevalent role in cervical cancer, whereas vaginal cancer can be caused by either (Figure 1) [59].

Radiation carcinogenesis is a third explanation for the link between vulva or cervix carcinoma and vaginal cancer [60]. The mechanism by which DES may contribute to the development of clear cell adenocarcinoma is unknown [61]. In a study of estrogen-induced maturation arrest of the Müllerian ducts, Robboy et al. proposed in 1984 that atypical cervical ectropion of the tuboendometrial type and atypical vaginal adenosis may be precursors to clear cell cancer of the vagina and cervix [62]. Postmenopausal women who use vaginal estrogen are at the same risk as women who do not use vaginal estrogen for invasive breast cancer, stroke, blood clots, endometrial cancer, and colorectal cancer [63].

### 2.5. Vulvar Cancer

Two distinct mechanisms lead to the development of vulvar cancer [64]. The occurrence of lichen sclerosis and differentiated vulvar intraepithelial neoplasia (dVIN) is one factor. This accounts for around 80% of vulvar cancers [65]. dVIN lesions and vulvar squamous cell carcinomas carry the same *TP53* mutations. The second form tends to affect teenagers who have been infected with high-risk HPV, most commonly HPV 16. The percentage of vulvar malignancies linked to HPV varies significantly between populations, spanning from 15–79%. Impaired DNA repair, variations in active repopulation signaling passageways, as well as impaired cell cycle regulation are also possible underlying causes [66].

EGFR is a transmembrane-receptor tyrosine kinase. When a ligand binds to EGFR, the receptor is autophosphorylated on tyrosine residues, activating a number of intracellular channels that can lead to cancer cell proliferation, metastasis, invasion, and promotion of tumor-induced neovascularization [67]. EGFR overexpression has been reported in 46–72% of patients with vulvar cancer [68]. A negative correlation between HPV status and EGFR amplification suggests that EGFR copy number increases play a role in vulvar carcinogenesis independent of HPV [69].

Angiogenesis is a critical step in tumor formation and metastatic spread. One of the most important controllers of this mechanism is the VEGF pathway [70]. In the case of vulvar cancer, VEGF overexpression is associated with high mortality and poor tumor distinction [71,72]. In VIN lesions, increased expression had also been reported, alongside VIN3 expressing much more than VIN1 and VIN2 (Figure 1) [73]. Vulvar cancers are rarely hormone-dependent tumors [74]. HRT is impartial in endometrial cancer type II, uterine carcinosarcoma, and adenosarcoma, some forms of ovarian cancer, cervical, vaginal, and vulvar squamous cell carcinoma, prolactinoma, kidney cancer, pancreatic cancer, and thyroid cancer [63]. However, tissue-selective estrogen complex incorporating BZA/CE is a possible menopause therapy for postmenopausal women [75].

## 3. Hormonal Therapy for Gynecological Cancers

### 3.1. Hormonal Therapy for Cervical Cancer

Hormonal therapy for cervical cancer has been reported in a variety of experimental and clinical investigations. Sawaya et al. reported that medroxyprogesterone acetate at a dose of 2.5 mg/day and oral conjugated equine estrogens at 0.625 mg/day were administered to 2561 women for two years. This study determined the predictive value of an abnormal cervical smear in postmenopausal women with recent normal smears. In the two years after a normal smear, the rate of new cytologic abnormalities was 110 per 4895 person-years (23 per 1000 person-years (95% CI, 18 to 27 per 1000 person years)). There was one case of mild to moderate dysplasia among the 103 women with documented histological diagnosis. The positive predictive value of any smear abnormality identified 1 year after a normal smear, therefore, was 0% (CI, 0% to 5.0%) (0 of 78 women); the positive predictive value of abnormalities found within 2 years was 0.9% (CI, 0.0% to 3.0%) (1 of 110 women). The findings concluded that the prevalence of cytologic abnormalities was non-significantly greater in hormone-treated women than in women who were not treated with hormone [76]. In postmenopausal women undergoing hormone replacement therapy (HRT), several studies found a significantly lower risk of cervical squamous cell carcinoma and a slight increase in the risk of adenocarcinoma. There are no indications that HRT has a negative effect on the oncological outcome of CC, and numerous advantages have been reported, such as improvement in quality of life and decreased metabolic risk, leading to the conclusion that HRT should be provided to young survivors of CC for the management of early menopause [77]. In another clinical trial, 645 women with cervical cancer aged 40–75 years were medicated with estrogen replacement therapy (ERT) and followed for ten years. The odds ratio was 0.9 (95% CI: 0.5–1.7). The odds ratios for cervical cancer decreased with duration of therapy when compared to a control group, decreasing to 0.6 (95% CI: 0.4–1.1) for timespans shorter than 12 months and 0.5 (95% CI: 0.2–1.0) for timespans of 12 months or longer. These results indicate that exogenous estrogens may reduce the incidence of cervical cancer [78]. K14E7 and K14E6 transgenic mice were treated with 0.15 mL of the ER antagonist Faslodex twice a week for one month, and 1.5 mg of raloxifene 5 days a week for one month. In this study, raloxifene, an ER antagonist and selective ER modulator, effectively cleared cancer and precursor lesions in both the vagina and the cervix [79]. The combination of synthetic estrogen and synthetic progestogen is effective in treating cervical cancer. After radiotherapy or surgery for stage I or II cervical cancer, 120 patients received 2 mg chlormadinone and 5 mg dienestrol daily for five years. The occurrence of cancer relapse was 20% in the group that underwent hormone treatment [80]. Hormonal therapy (HT) consisting of progesterone, estrogen, or progesterone and estrogen together was applied in 804 CIN3/CIS cases in peri- and postmenopausal women and 261 invasive cervical cancer (ICC) cases in three distinct groups of women who received treatment for different time periods, ≤1, 2–4, and ≥5 years. The therapy was found to be associated with a lower risk of ICC (HR = 0.5, 95% CI: 0.4–0.8). The use of HT by peri- and postmenopausal women reduced the risk of ICC substantially. However, estrogen alone was associated with a higher incidence of CIN3/CIS, whereas coupled HT was associated with a lower incidence of ICC [81]. 

**Figure 2:**
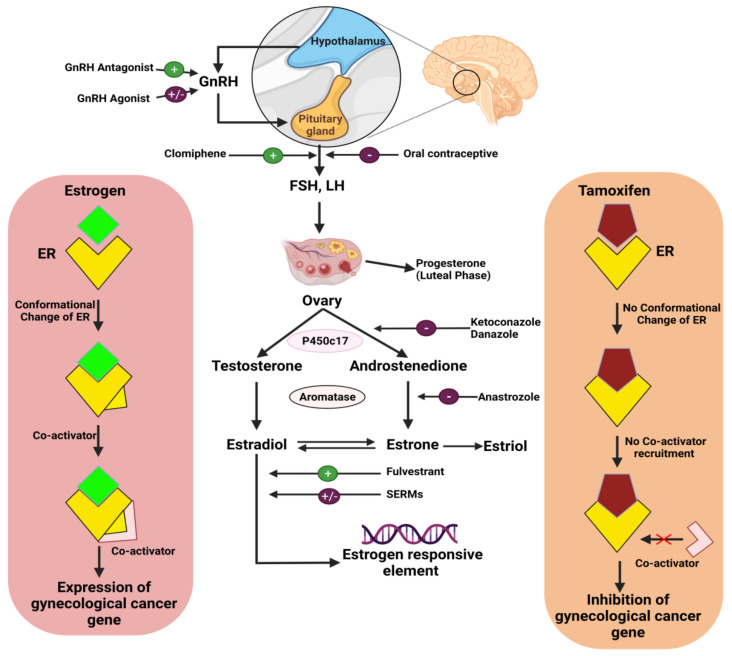
Hormonal therapies to counter gynecological cancer progression.

Certain cervical cancers may respond in a hormone-independent manner to a combination treatment consisting of triphenylethylene and the antiestrogen medication tamoxifen. Roig et al. conducted an experiment on 19 patients with invasive cervical cancer and found that tamoxifen treatment for 10 days at 20 or 40 mg/day resulted in alterations in growth and distinction levels in some cervical carcinomas [82]. Another study by L. Bigler found that 10 mg of the antiestrogen medication tamoxifen taken twice a day orally in 34 non-squamous cell carcinoma patients with median age of 49 had an objective response rate of 11.1% [83]. Another trial studied the use of progestagen mixed oral contraceptives in 16,573 women who had cervical cancer and 35,509 who did not. The cohort was split into groups that had used progestagen for fewer than five years, and groups that had used progestagen for more than five years. The risk of cervical cancer was increased in the groups with over five years progestagen use [84]. Progesterone, estradiol, and estrone are essential hormones that are utilized as treatments for cervical cancer. In one study, 11,742 women aged over 18 were subjected to HT, including progesterone (100 pg·mL^−1^), oestrone (5 pg·mL^−1^), SHBG (20 nmol L^−1^), oestrone sulphate (100 pg·mL^−1^), estradiol (5 pg·mL^−1^), and DHEAS (10 μg·dL^−1^). After observation for 5–10 years, it was found that increased plasma levels of progesterone or endogenous estrogens reduced the incidence of cervical neoplasia (Figure 2) [85].

Pannoneon and Rauh conducted a clinical trial including 222 women. After treatment for cervical cancer, 48% of patients received HRT counseling and efforts to decrease inequities in the provision of survivorship care were enhanced (Table 1) [86]. Tamoxifen at 0, 1, 2.5, 5, 7.5, or 10 μM for a time span of 6 days suppressed the in vitro proliferation of three cervical carcinoma cell lines generated from uterine cervical carcinoma (ME-180, CaSki, and HeLa). Progressive cell death and cytotoxicity were found at doses greater than 5 μM. A dose of 2.5 μM tamoxifen inhibited development by more than 60% in the CaSki cell line, while 5 μM tamoxifen was cytotoxic [87].

Everhov reported that estrogens, contraceptives, progestogens, and estrogen–progestogen combination are potent treatments for cervical cancer. In their experiment it was found that 171 (67%) of 257 women had at least one course of HT for half a year to a whole year after the diagnosis. Slightly less than half of cervical cancer survivors who had therapy-induced early menopause underwent HT at or close to the suggested dose while reducing the dosage over time. This implies that HT can be safely given to survivors of cervical cancer [88]. Nevertheless, a study of women aged 15–49 taking ethinylestradiol (30–35 μg) between 1995 and 2014 reported that the risk of cervical cancer increased during ethinylestradiol usage and decreased after cessation [89].

### 3.2. Hormonal Therapy for Ovarian Cancer

Since the etiology of ovarian cancer is poorly understood, primary prevention efforts are difficult. According to a meta-analysis of data covering the years 1966–2006, postmenopausal use of HT increased the risk of ovarian cancer by 30%, and estrogen therapy (ET) alone carried a higher risk than estrogen plus progestin therapy (EPT) [91]. Hormone treatment for ovarian cancer patients usually consists of a combination of medications that suppress estrogen levels in the body. Hormone treatment can also be used to raise progesterone levels, which might inhibit cancer cell growth in some circumstances [44]. The most common treatments for ovarian stromal tumors are hormone-inhibiting or hormone medications. Leuprolide and goserelin are two examples of LHRH agonists [92]. Tamoxifen is a drug that can be used to treat ovarian stromal tumors, but is only utilized in rare cases to manage advanced ovarian epithelial cancers. The purpose of tamoxifen therapy is to prevent endogenous estrogens from triggering cancer cell proliferation [93]. Exemestane, anastrozole, and letrozole are aromatase inhibitors that prevent estrogen synthesis. As they do not block ovarian estrogen production, they are only useful for reducing estrogen levels in postmenopausal women (Figure 2) [94].

According to the findings of a global randomized medical study, women who received estrogen hormone replacement treatment (HRT) following diagnosis with epithelial ovarian cancer survived longer than women who did not receive estrogen (Figure 2) [95]. The gonadotropin-releasing hormone analog leuprolide acetate (1 mg) was administered to 23 patients with refractory epithelial ovarian cancer for a minimum of 8 weeks, providing evidence of antitumor activity against refractory grade 1 epithelial ovarian adenocarcinoma [96].

When used during CA125 relapses, the aromatase inhibitor letrozole can result in disease stability and CA125 responses, which are associated with increased ER expression. Sixty patients were given 2.5 mg letrozole daily for 12 weeks, and the findings showed that future gynecological cancer investigations might focus on a group of endocrine-sensitive aromatase inhibitors [97]. Similarly, in a group of 27 patients, letrozole at a dosage of 2.5 mg once daily was shown to be an effective and safe treatment for recurrent ovarian cancer [98]. Veenhof also noted the efficacy of hormone treatment (Table 2) [99]. Ovarian cancer cells were shown to be resistant to megestrol acetate, a kind of progestin hormone treatment. In 72 patients, megestrol acetate 800 mg/day for one month preceded by 400 mg/day as a maintenance dose was given, with results demonstrating that increasing the dose did not increase the all-inclusive 10% benefit of hormone therapy for chemotherapy-resistant ovarian cancer [99].

Another post-M.D. study investigated the effects of progesterone and estrogen on 2933 women with advanced epithelial ovarian cancer. For advanced serous ovarian cancers and endometrioid carcinoma, ER and PR are predictive indicators. In this study, PR expression was associated with improved disease-specific survival in endometrioid carcinoma (log-rank *p* < 0.0001) and high-grade serous carcinoma (log-rank *p* = 0.0006), and ER expression was associated with improved disease-specific survival in endometrioid carcinoma (log-rank *p* < 0.0001) [100]. John reported that 12 weeks of treatment with a combination of 2.5 mg twice daily letrozole and 10 mg twice daily everolimus in 20 patients led to a 47% progression-free survival with tolerable toxicity in individuals with ER-positive recurrent advanced ovarian cancer [101].

Estrone sulfate (E1S) is used to treat gynecological cancers. After 15 and 60 min incubation times, 12 postmenopausal women with non-estrogen-producing ovarian tumors were given 20 pmol of [3H] E1S in 100 mL of Tris-HCl. According to Dmitrijus, in postmenopausal women with non-estrogen-producing ovarian tumors, tumor tissue transformation of circulating E1S to E2 might be one of the major causes of high S-E2 levels [102]. However, the injection of a single dose of gonadotropin daily in 100 infertile clomiphene citrate-resistant women with PCOS appeared to be more successful at inducing ovulation [103].

### 3.3. Hormonal Therapy for Uterine Cancer

Endometrial cancer is hormone-dependent and is usually preceded by endometrial hyperplasia. A variety of histologic subgroups have been identified, each with its own natural history. Although no link has been found between molecular profile, histology, and hormone response, the underlying biology has been discussed [102]. Hormonal treatments for endometrial cancer include tamoxifen, GnRHa, aromatase inhibitors (AIs), progestins, and luteinizing hormone-releasing hormone agonists (LHRH agonists) (Figure 2) [105].

#### 3.3.1. Progestins

Progesterone is an endometrial hormone that inhibits estrogen-induced growth. Though endometrial neoplasias can decline in response to progestin therapy, this is not always the case [56]. Progesterone or medications similar to it (progestins) are the most common hormone treatments for endometrial cancer. The two most commonly used progestins are megestrol acetate, which is taken as a tablet or a fluid, and medroxyprogesterone acetate, which is administered as an injection or a tablet. The proliferation of endometrial cancer cells is slowed by these medications. They have been found to be beneficial in the treatment of women who have endometrial cancer and desire to conceive in the future [106]. Stromal proliferation and the endometrial gland are both reduced by progestins. They can reduce cyclin-dependent kinase (CDK) expression and increase the expression of an endogenous CDK inhibitor, p27, preventing CDKs from binding to cyclins and blocking cell cycle progression, resulting in cell growth suppression. Medroxyprogesterone acetate (MPA) and megestrol acetate (MA) are two of the most regularly utilized progestins (Figure 2) [107,108].

The use of estrogens for a long duration without progestins, or with the periodic addition of progestins, increases the risk of endometrial cancer. To reduce the risk of endometrial cancer associated with estrogen replacement treatment, progestins may need to be given on a regular basis. Medications containing only estrogens were linked to a significant increase in endometrial cancer risk, which increased with time and dosage [109]. In order to successfully prevent the heightened risk of endometrial cancer, the progestin in successive estrogen–progestin replacement treatment must be administered for at least 10 days. The use of a continuous estrogen–progestin combination is also successful. Neither treatment lowers a woman’s risk of endometrial cancer. The stark difference in the impacts of progestin usage for 10 or more days (viably 10 days) compared to less than 10 days (viably 7 days) in sequential estrogen–progestin replacement therapy implies that the degree of endometrial sloughing may be a key factor in assessing endometrial cancer risk [110].

#### 3.3.2. GnRHa

GnRHa is a synthetic GnRH analog with increased half-life, stability, and affinity for the GnRH receptor. GnRHa competes with GnRH for the binding site of the GnRH receptor, inhibiting Gn release from the hypophysis and lowering estrogen levels. When GnRH or GnRHa levels are raised, LH and follicle-stimulating hormone (FSH) levels rise quickly [111]. It was shown that the GnRH receptor is expressed in approximately 80% of ESS, implying that GnRHa may be activated by inhibiting these GnRH-R [112]. Furthermore, studies in vivo and in vitro [113,114] showed medication with standard doses of GnRH agonists, which restrict ovarian estrogen production and pituitary gonadotropin release, have become a part of early stage EC or pre-cancer (atypical endometrial hyperplasia) fertility preservation treatment. In severe or recurring EC, typical doses of GnRH agonists demonstrated only minimal activity. Clinical trials using higher doses or stronger analogs, such as GnRH-II antagonists, have yet to be conducted. In a phase II study, the GnRH analog zoptarelin doxorubicin, which is cytotoxic, showed promising effects in patients diagnosed with recurrent or advanced EC that expressed GnRH-R. The cytotoxic GnRH doxorubicin compound was no better than free doxorubicin in a phase III study in patients who had EC with undetermined GnRH-R expression. Future clinical studies focusing on the GnRH system in EC with better planning and design might be beneficial [115].

#### 3.3.3. Aromatase Inhibitors

Aromatase inhibitors, which have been shown to reduce the risk of breast cancer and levels of endogenous estrogen, may also minimize the risk of developing endometrial cancer [116]. In almost 60% of uLMS and 80% of LGESS, aromatase was expressed in the tumor. Aromatase inhibitors also inhibit both the production of estrogen inside tumor cells and the amount of estrogen in the blood by decreasing the action of aromatase in peripheral tissues [117,118]. For the clinician, metastatic/recurrent endometrial adenocarcinoma that is resistant to local or regional treatment or/and chemotherapy is a disheartening clinical entity. A 58-year-old woman with persistent endometrial cancer that was resistant to treatment with chemotherapy was effectively treated with anastrozole, an aromatase inhibitor [119].

#### 3.3.4. SERMs

SERMs are a class of estrogen receptor modulators that include the nonsteroidal drugs toremifene and tamoxifen, as well as the steroidal fulvestrant. Tamoxifen and toremifene have been shown to reproduce the action of estrogen in the uterus, stimulating the growth of endometrial cancer [120,121]. Hormonal treatment has been shown to be beneficial for metastatic, recurrent, or unresectable low-grade endometrial stromal sarcoma (LGESS) and hormone receptor-positive (ER+/PR+) uterine leiomyosarcoma (uLMS) with good tolerance and cooperation. Hormonal medication can be further utilized to preserve fertility in people with early stage cancer [55]. Low-grade endometrial stromal sarcoma (EES) is an uncommon tumor with high recurrence but favorable prognosis. Case studies have documented how these recurrences respond to hormone therapy [122].

Anastrozole (1 mg/day) combined with letrozole (2.5 mg/day) and exemestane (25 mg/day), both of which are aromatase inhibitors, can be used to treat uterine cancer. Patients who had uterine sarcoma, including four patients with ESS and three patients with LMS, were treated for 29.2 months with this medication, demonstrating that aromatase inhibitors are helpful in the treatments of ESS [117]. Ramirez treated 81 individuals with either megestrol acetate (28 patients; 35%), medroxyprogesterone acetate (36 patients; 44%) or progestins for 24 weeks. Most patients with well-distinguished endometrial cancer are treated with progestins in a cautious manner. Carcinoma expansion outside of the uterus is uncommon when response to progestins is not obtained or when the illness recurs [123].

A combination of endogenous or exogenous progestins and estrogen, as well as 160 mg megestrol acetate, is often a beneficial hormonal treatment for uterine cancer. Chu and Mor reported that ERT may be harmful in patients with low-grade ESS, based on a study of 22 patients, in which hormonal treatment was given for an average of 100 months (range, 2–258). Reduction of ERβ might be a sign of cancer. Adjuvant progestin medication and the treatment of recurring ESS should indeed be routinely evaluated [124]. In another D. Pink trial, tamoxifen, ERT, progestin, and aromatase inhibitors were administered together for durations of 4–164 months in uterine cancer patients. Individuals with a prior record of low-grade ESS ought not to be medicated with tamoxifen or estrogens, according to the results of a sarcoma database that was checked for all occurrences of metastatic ESS detected since 1999, including approximately 800 patients. Letrozole and MPA are extremely efficient and, in most cases, result in long-term illness stabilization [120]. Furthermore, 13 women were given MA and letrozole for 4–252 months (median 48 months). For patients with significant recurrent or residual low-grade ESS, hormonal therapy with aromatase inhibitors and progestin had good reliability and should be regarded as the preferred treatment if recurring illness cannot be effectively resected [122].

Mizuno reported that medroxyprogesterone acetate (MPA), a treatment that uses progesterone, was used on 13 successive patients with low-grade ESS for an average of 64 months (ranging from 28–92 months). During the 117-month follow-up timeframe, MPA treatment for recurrent or residual illness resulted in good control of disease over a period of 5 years. These findings suggest that MPA treatment could be explored as a possible treatment for persistent or recurrent low-grade ESS [125]. In another study, 40 patients with advanced uLMS were treated with aromatase inhibitors such as letrozole (in 74% of patients), exemestane (in 6% of patients), and anastrozole (in 21% of patients), with only 9% obtaining an objective response. Patients who had ER+ and/or PR+ tumors had a longer progression-free survival than those with ER− and PR− tumors [126].

Aromatase inhibitors and progestin were used in the case of a 48-year-old woman identified with stage I ESS. The patient was given three rounds of bleomycin, etoposide, and cisplatin (BEP), megestrol acetate, and anastrozole, and was monitored for two years. ESS can be healed with aromatase inhibitors and progestin, according to the findings, since ESSs with a sex-cord stromal component are hormonally functional [127]. Murphy reported a patient with an enlarged uterus, dysmenorrhea, and menorrhagia who was medicated with adriamycin, GnRH agonist, ifosfamide, leuprolide acetate, and cisplatin and monitored for 15 months following diagnosis. The symptoms of leiomyosarcomas, such as rapidly increasing uterine masses, pelvic discomfort, and uterovaginal hemorrhage, are disguised by GnRH medication. However, in this case, the latency in surgical treatment during GnRH administration resulted in a negative outcome (Table 3) [128].

The efficacy of tamoxifen and megestrol acetate, hormonal treatment with progestins and estrogen, and GnRHa in uterine cancer is well established (Figure 2). A combination of GnRHa, tamoxifen (30 mg/day), and megestrol acetate (160 mg/day) was given to 9 patients with confirmed stage IA, grade 1 endometrial adenocarcinoma. After treatment for 6 months, 8 (88.9%) of the 9 patients achieved full recovery. All nine of the patients have been disease-free for 25–113 (median 69) months since their first diagnosis [129]. In a study by Fu, a 22-year-old nullipara was treated with tamoxifen and megestrol acetate for 6 months and 1 year respectively after curettage, and there was no sign of illness [130]. Shih and Jung reported that megestrol (1 month), tamoxifen (20 mg/day), and depot leuprolide acetate subcutaneous injection (3.75 mg/month) were used to treat a 36-year-old nulliparous woman with stage IA, grade 1 endometrial cancer. They concluded that adverse clinical examinations are not encouraging for a recurrent low-grade, early-stage endometrial adenocarcinoma without conventional hormonal therapy [131]. In two women of reproductive age, 500 mg of oral medroxyprogesterone was administered for 6 months and twice weekly for 9 months. With medroxyprogesterone medication, the quarterly timeframe for D&Cs was acceptable, and the patients’ wishes to avoid hysterectomy were fulfilled. As a result, the progestin and estrogen-based hormone therapy proved effective in the treatment of uterine cancer [132]. Endometrial cancer prevalence was reduced in women on an aromatase inhibitor in comparison to those taking tamoxifen in a peer group-based, coordinated health care plan system. Aromatase inhibitors may also help to reduce the risk of tamoxifen-related endometrial cancer. Although the aromatase inhibitor group had somewhat fewer endometrial malignancies than the no treatment group, further research is required to evaluate this possible relationship [116].

### 3.4. Hormonal Therapy for Vaginal Cancer

Vaginal carcinoma is a rare gynecologic malignancy. Surgery can be used to treat vaginal carcinoma in situ and extremely early stage invasive cancer of the vagina. Radiation therapy is the usual treatment for women with vaginal cancer. To maintain the structure and functionality of the vagina, radiotherapy is used as a treatment of last resort for early phase vaginal cancer. Radiation therapy is used to prevent exenterative surgery, maintain function and anatomy, and address identified or suspected lymph node metastases in later stages of vaginal cancer. Estrogen (estrone sulfate) in the form of vaginal promestriene (10 mg soft vaginal suppository) was given to 17 postmenopausal women daily for one to six months to treat vaginal cancer. The amount of circulating E1S in extremely symptomatic gynecological cancer patients was not influenced by vaginal promestriene treatment, although there was a broad range of individual values before and after treatment [66].

To investigate ElS as a hormonal treatment, the AC-258 cell line and the squamous vaginal carcinoma cell line EC-82 were treated with ElS and DS (2.25 μCi, 15 μM) for at least two hours using cell densities of up to 3.2 × 10^6^ cells/mL. In situ estrogen synthesis from steroid sulfate antecedents in the organs and tissues of and around the reproductive tract may influence the growth of ovarian and vaginal tumors [134]. Meanwhile, in a 52-week double-blind, randomized, placebo-controlled trial, 205 postmenopausal women were given an ultra-low-dose 10 μg 17-estradiol vaginal tablet, which did not increase the incidence of endometrial carcinoma or hyperplasia [135]. A study of 423 women who were given local estrogen therapy in the form of either estradiol cream (0.1 mg estradiol/g USP) or estradiol vaginal tablets (10 g) reported that vaginal tablets have higher compliance than vaginal cream, with the majority of respondents preferring vaginal tablets [136]. The impact of estrogen on vaginal cancer was also reported in a study in which the estrogen receptor modulator ospemifene was given for one month to 32 postmenopausal women receiving surgical intervention, with the results demonstrating that ospemifene improved the signs of atrophy and cancer by enhancing ERα expression and maturation of the vaginal mucosa [137]. Bachmann reported that 30 or 60 mg/day ospemifene for 12 weeks was efficacious for the treatment of vaginal dryness and dyspareunia (that can progress to vulvovaginal malignancy and atrophy if left untreated) but also better received in 826 postmenopausal women (Table 4) [138].

### 3.5. Hormonal Therapy for Vulvar Cancer

Jacob reported that HRT with progesterone and estrogen in a population of 7189 women was not harmful. The majority of vulvar malignancies in this cohort were estrogen-independent squamous cell carcinomas [139]. The estrogen receptor modulator ospemifene was found to be helpful for the prevention of vaginal and vulvar cancer and atrophy in women with dyspareunia in a study of 605 postmenopausal women using oral ospemifene 60 mg/day for 12 weeks [140].

The estrogen receptor modulator bazedoxifene (BZA) was given to 664 postmenopausal women for 12 weeks at doses of 20 mg/CE, 0.625 mg, 0.45 mg, and 20 mg. BZA/CE was efficacious in preventing mild-to-advanced VVA and vaginal symptoms. These findings suggest the use of a BZA/CE-containing tissue-selective estrogen complex as a potential menopause treatment for postmenopausal women [141]. Conjugated estrogens 0.625 mg and 0.45 mg BZA 20 mg were administered to 2 groups of 1583 postmenopausal women and provided endometrial protection without increasing breast pain/density, vaginal bleeding, or ovarian cysts after 2 years of observation [75]. Additionally, in 652 symptomatic postmenopausal women, therapy with BZA/CE for a duration of 12 weeks dramatically enhanced quality-of-life parameters and attenuated cancer [142]. Following a 12-week study of 25 women with endometrial biopsies, Mirkin speculated that 4 μg and 10 μg vaginal estradiol (E2) does not cause endometrial hyperplasia resulting in moderate to severe dyspareunia, which is also considered to be a symptom of vaginal and vulvar cancer and atrophy (Table 5) [143].

## 4. Risk Factors Associated with HRT in Gynecological Cancers

Hormone treatment slows or prevents the growth of hormone-sensitive cancers by interacting with the actions of hormones on breast cancer cells or by limiting the body’s ability to produce hormones [145]. Postmenopausal women who received “some” estrogen treatment are more likely to develop estrogen-receptor and progesterone-receptor positive breast tumors [146]. The application of antiestrogenic drugs such as nafoxidine and tamoxifen has shown to be the most effective new hormonal treatment for estrogen receptor-positive metastatic breast cancer. Both of these medications have anticancer effectiveness similar to other additive hormonal treatments, and they are better tolerated due to the lack of significant toxicity [147]. The therapeutic uses of aromatase inhibitors (AI) and GnRH agonists that limit estrogen production, tamoxifen, a selective estrogen receptor modulator (SERM), and fulvestrant, a selective ER downregulator (SERD) have resulted in significant improvements in survival outcomes for patients with ER+ breast cancer via ER-alpha (ESR1) mutation, growth factor receptor signaling, as well as PI3K/Akt/mTOR, CDK4/6, and epigenetic and immunological checkpoints as resistance mechanisms [148]. HRT may raise the risk of developing heart disease, stroke, and type 2 diabetes and inflammatory markers (such as C-reactive protein) [149,150]. HT may decrease stiffness of the aorta and large arteries in postmenopausal women, with potential benefit for age-related cardiovascular disorders. The reduction of arterial compliance with age appears to be altered with hormonal therapy [151].

The effects of hormone treatment against invasive ductal, lobular, and tubular carcinoma were typically larger for estrogen-progestagen therapy and lessened with rising BMI (body mass index) [152]. Tamoxifen use highly increased the incidence of secondary diabetes, although aromatase inhibitors had no effect on diabetes mellitus in individuals with initial breast cancer [153]. However, HT is a recognized risk factor for blood clots like deep vein thrombosis or pulmonary embolism [154]. Moreover, HT also raises liver disease and unusual vaginal bleeding that has not been reviewed by physicians [155]. The increased risk is related to how long the HRT is administered, and it falls after the patient stops taking it [156].

## 5. Concluding Remarks and Future Directions

HT is a promising therapeutic option for patients with recurring gynecological malignancies, whose primary goal is palliation and life extension (rather than cure). Aromatase inhibitors can be used for long periods of time with even less cumulative injury as these inhibitors are usually well tolerated. Previous studies have reported a wide range of functional properties and response rates. HT is often thought of as “less potent” in comparison to chemotherapy, yet it can be just as useful in certain types of gynecological cancer. Hormone treatment is classified as a “systemic” therapy since it affects the entire body. In addition to gynecological cancer prevention, HRT has shown potential in treating postmenopausal symptoms. HRT, such as progestin and estrogen combination therapy or estrogen monotherapy has been shown in recent trials to benefit postmenopausal women with osteoporosis as well as some concomitant disorders. HT can stop or slow the growth of cancer, and reduce the chance that it will return. To promote a better future generation and healthy life, HT for gynecological illnesses is very important. Further study and clinical trials should be undertaken to discover new benefits of HT for gynecological cancers.

## Figures and Tables

**Table 1:** Experimental and clinical studies of hormonal therapy to treat cervical cancer.

Name of Hormone	Formulation Name and Dose	Observation Time	Study Model	Results	References
ER antagonist	0.15 mL Faslodex and 1.5 mg of raloxifene	Faslodex twice a week for a month and raloxifene for a month, 5 days a week	K14E7 and K14E6 transgenic mice	Raloxifene, ER antagonist and selective ER modulator, efficiently clears cancer and its precursor lesions in both the cervix and the vagina	[79]
Non-steroid synthetic estrogen with synthetic progestagen	Dienestrol—1 tablet (5 mg) and Chlormadinon—1 tablet (2 mg)	5 years	120 patients after surgery and/or radiotherapy	Only 20% and 32% incidence of cancer recurrences and survival without cancer symptoms was found in 80% and 65% of cases, respectively in the hormonally treated group and in the control group	[90]
Estrogen and progesterone	HT formulation (estrogen alone, progesterone alone, combination of estrogen/progesterone)	3 groups (≤1, 2–4, ≥5 years)	261 ICC and 804 CIN3/CIS cases of post- and perimenopausal women	Significantly decreased the risk of ICC in peri- and postmenopausal women, but menopausal estrogens alone were associated with an increased risk of CIN3/CIS and combined HT was inversely associated with ICC	[81]
ERT	—	1–10 years	645 women	Exogenous estrogens decreased the risk of cervical cancer	[78]
Estrogens progestin	Oral conjugated equine estrogens, 0.625 mg/day, plus medroxyprogesterone acetate, 2.5 mg/day	2 years	2561 women	Did not significantly affect the incidence of cytologic abnormalities	[76]
Anti-estrogen	Triphenylethylene antiestrogen tamoxifen	10 days (20 or 40 mg/day)	19 patients	Certain cervical carcinomas had changes in their proliferation and differentiation levels following tamoxifen administration	[82]
Anti-estrogen	Tamoxifen	10 mg per orally twice a day	34 patients	The objective response rate was 11.1%, so tamoxifen appears to have minimal activity in non-squamous cell carcinoma of the cervix	[83]
Progestagen	Combined oral contraceptives	<5 years and >5 years	16,573 women	The risk of invasive cervical cancer increased with increasing duration of use, not for short time use	[84]
Estrone, estradiol and progesterone	SHBG (20 nmol·L^−1^) estradiol (5 pg·mL^−1^) estrone (5 pg·mL^−1^) estrone sulphate (100 pg·mL^−1^) DHEAS 10 μg·dl^−1^ and progesterone 100·pg·mL^−1^	5–10 years of study	11,742 women	Elevated plasma levels of endogenous estrogens or progesterone decrease the risk of cervical neoplasia	[85]
HRT	—	1 January, 2005 to 31 December 31 2015	222 women	48% patients received counseling for HRT and then improved efforts to reduce disparities in the distribution of survivorship care	[86]
ER modulator, Tamoxifen	5% dextran-charcoal treated fetal bovine serum (D5) and 0, 1, 2.5, 5, 7.5, or 10 μM tamoxifen	6 days	In vitro growth of three cell lines derived from carcinoma of the uterine cervix (HeLa, CaSki, ME-180)	Inhibited cell growth of the cervical carcinoma cell lines; 2.5 μM tamoxifen induced more than 60% growth inhibition where 5 μM tamoxifen was cytotoxic	[87]
Estrogens and progestogens	Contraceptives (G03A), estrogens (G03C), progestogens (G03D), and progestogens and estrogens in combination (G03F).	0.5 to 1 year after diagnosis	171 (67%) of 257 women had at least one dispensing of HT (Hormonal Therapy)	Fewer than half of cervical cancer survivors with therapy-induced early menopause used HT	[88]
Estradiol	30 to 35 μg ethinylestradiol	From 1995 to 2014	women aged 15 to 49	The risk pattern among any hormonal and combined contraceptive users generally increased with longer duration of use and declined after stopping	[89]

**Table 2:** Experimental and clinical studies of hormonal therapy to treat ovarian cancer.

Name of Hormone	Formulation Name and Dose	Observation time	Study Model	Results	References
GnRHa	Leuprolide acetate (1 mg)	8 weeks	23 patients	Showed evidence of antitumor activity against refractory grade 1 epithelial adenocarcinoma of the ovary	[96]
Aromatase inhibitor	Letrozole (2.5 mg daily) at the time of CA125 relapse	12 weeks	60 patients	Produced disease stabilization and CA125 responses that in turn are linked to higher levels of ER expression	[97]
Aromatase inhibitor	Letrozole at a dose of 2.5 mg once a day		27 patients	The aromatase inhibitor letrozole is an agent with some activity and limited toxicity for relapsed ovarian cancer.	[98]
Progestin	Megestrol acetate: 800 mg/day for 1 month followed by 400 mg/day as maintenance treatment	1 month	72 patients	This study does not suggest that the overall 10% benefit from hormonal therapy for chemotherapy refractory ovarian cancer will improve by increasing the dose	[99]
Estrogen, progesterone	-		2933 women	PR expression and ER expression were associated with improved disease-specific survival	[100]
Aromatase inhibitor	Oral everolimus 10 mg daily and letrozole 2.5 mg daily	12 weeks of therapy with the combination of everolimus and letrozole	20 patients	Associated with a promising (47%) progression-free survival rate in patients with ER-positive relapsed high-grade ovarian cancer	[101]
Gonadotropin	Injection of single dose gonadotropin daily	2003–2008	100 infertile clomiphene-citrate resistance women with POCS	Pregnancy and abortion rate in infertile women of PCOS receiving gonadotropin as a treatment for induction of ovulation seems to be more effective	[103]
E1S	20 pmol of [3H] E1S in 100 mL of Tris-HCl	(15 and 60 min) incubation time	12 postmenopausal women	Conversion of circulating E_1_S to E_2_ by the tumor tissue could be one important reason for elevated S-E_2_ levels in postmenopausal women with non-estrogen-producing ovarian tumors	[104]

**Table 3:** Experimental and clinical studies of hormonal therapy to treat uterine cancer.

Name of Hormone	Formulation Name and Dose	Observation Time	Study Model	Results	References
Aromatase Inhibitor	Anastrozole (1 mg/day), exemestane (25 mg/day)-1 patient, letrozole (2.5 mg/day)	29.2 months	Patients with uterine sarcoma (4 patients with ESS, endometrial stromal sarcoma, and 3 patients with LMS)	Effective in the treatment of endometrial stromal sarcomas	[117]
Progesterone	Medroxy progesterone acetate (36 patients; 44%) or megestrol acetate(28 patients; 35%), progestins	24weeks	81 patients	When disease recurs, carcinoma extending beyond the uterus is rare in patients reported with well-differentiated endometrial adenocarcinoma who undergo treatment with a progestational agent	[123]
Estrogen, progestin, aromatase inhibitors	ERT, tamoxifen, progestins, aromatase inhibitors	4 to 164 months	800 patients	MPA and letrozole, in particular, are highly effective and lead to sustained disease control in most cases	[120]
Progestin, Aromatase inhibitors	Megestrol acetate (MA), Aromatase inhibitor (letrozole)	4+ to 252+ months (median 48+ months).	11 patients	Hormonal treatment for measurable residual or recurrent low-grade ESS has a high response rate and should be considered as the treatment of choice for patients in which recurrent disease cannot easily be eliminated	[122]
Exogenous or endogenous estrogen and progestins	Megestrol acetate 160 mg, progestins	100 months (range, 2–258)	22 patients	ERT was detrimental in patients with low-grade endometrial stromal sarcoma, but progestin therapy should be routinely considered for adjuvant therapy and for the treatment of recurrent endometrial stromal sarcomas	[124]
Progesterone	Medroxy progesterone acetate (MPA)	Dosing period 64 months (range 28–92 months) but follow-up period was 117 months	13 patients	MPA therapy might be considered as a therapeutic option for residual or recurrent low-grade ESS and perhaps chosen as a first-line therapy	[125]
Aromatase	Aromatase inhibitors used were letrozole (in 74% of patients), anastrozole (21%), and exemestane (6%)	Between 1998 and 2008	40 patients	Aromatase inhibitors achieved objective response in only 9%. Progression free survival was longer among patients with ER and/or PR positive tumors than among patients with ER and PR negative tumors	[126]
GnRH agonist	GnRH agonist, leuprolide acetate, adriamycin, cisplatin, ifosfamide etc.	15 months	A patient with menorrhagia, dysmenorrhea, and an enlarged uterus	GnRH therapy mask the symptoms of leiomyosarcomas, e.g., rapidly enlarging uterine mass, pelvic pain, uterovaginal bleeding	[133]
Progestin and aromatase	Three cycles of BEP (bleomycin, etoposide, cisplatin), anastrozole and megestrol acetate	2 years	48-year-old woman was diagnosed with stage I endometrial stroma sarcoma	Endometrial stromal sarcoma with sex-cord stromal component may be hormonally functional and can be cured by treating with progestin and aromatase inhibitor	[127]
Estrogen, progesterone	Megestrol acetate and tamoxifen	6 months	A 22-year-old nullipara	1 year after the last curettage, there is no evidence of disease	[130]
Estrogen, progesterone	Combinations of megestrol acetate (160 mg/day), tamoxifen (30 mg/day), and GnRHa	6 months	9 patients with clinically diagnosed endometrial adenocarcinoma stage IA, grade 1	Of the 9 patients, 8 (88.9%) achieved complete remission after hormone therapy. All nine patients have been alive without evidence of disease	[129]
Estrogen, progesterone, (GnRHa)	Megestrol (1-month), tamoxifen (20 mg/day) and depot leuprolide acetate subcutaneous injection (3.75 mg/month)	6-months	A 36-year-old nulliparous woman	This case report signals a warning that negative clinical investigations are not reassuring for a relapsing endometrial adenocarcinoma failing conservative hormonal treatment	[131]
Estrogen, progestin	500 mg of oral medroxyprogesterone for 6 months, twice weekly	9 months (range, 3–18 months)	2 women	The quarterly interval for D&Cs was satisfactory with medroxyprogesterone treatment, and the patients’ desire not to undergo hysterectomy was met	[132]

**Table 4:** Experimental and clinical studies of hormonal therapy to treat vaginal cancer.

Name of Hormone	Formulation Name and Dose	Observation Time	Study Model	Results	References
Estrogen (estrone sulfate)	Vaginal promestriene 10 mg soft vaginal suppository daily for one month	1–6 months	17 patients (after menopause)	The level of circulating E1S was not significantly affected by vaginal promestriene treatment, but a wide range of levels was noted pre- and post-treatment in individual patients	[66]
E1S	ElS and DS (2.25 μCi, 15 μM)	Incubation time for at least 2 h and with cell number up to 3.2 × 10^6^ cells/mL	AC-258 cell line, squamous vaginal carcinoma cells (line EC-82)	Played a role in the development of tumors of the ovary and vagina	[134]
Estradiol	Ultra-low-dose 10-microgram 17β-estradiol vaginal tablets	52 weeks	(*n* = 205) in a randomized, double-blind, placebo-controlled trial	There was no increased risk of endometrial hyperplasia and carcinoma in postmenopausal women	[135]
Estrogen	Local estrogen therapy (LET) in form of estradiol vaginal tablets, 10 μg, estradiol cream, 0.1 mg estradiol/g USP	5-week period, from 6 March 2012 through 9 April 2012	423 women	There was greater compliance with vaginal tablets than with vaginal cream; respondents preferred their current treatment with the vaginal tablet	[136]
Estrogen	ER modulator (ospemifene)	1 month	32 postmenopausal women	Increased maturation, and ERα expression of the vaginal mucosa. These changes partially explained the improvement of symptoms of vaginal atrophy and cancer	[137]
ER modulator	Ospemifene 30 or 60 mg/day	12 weeks	826 postmenopausal women were randomized	Effective and well tolerated for the treatment of the symptoms of vaginal dryness and dyspareunia associated with vulvovaginal atrophy over	[138]

**Table 5:** Experimental and clinical studies of hormonal therapy to treat vulvar cancer.

Name of Hormone	Formulation Name and Dose	Observation Time	Study Model	Results	References
Estrogen, progesterone	Hormone replacement therapy	12 months	7189 women	Significantly decreased the proportion of women receiving at least one HRT prescription. The majority of vulvar cancers are not estrogen-dependent, and the prescription of HRT is not contraindicated after the diagnosis of this type of cancer	[139]
ER modulator	Oral ospemifene 60 mg/day	12 weeks	605 women	Effective for the treatment of vulvar and vaginal atrophy and cancer in postmenopausal women with dyspareunia	[140]
ER modulator	BZA 20 mg/CE 0.625 mg and 0.45 mg, BZA 20 mg	12 weeks	Healthy postmenopausal women (*n* = 664; aged 40–65 y)	Effective in treating moderate to severe VVA and vaginal symptoms.	[141]
Estrogen and ER modulator	Conjugated estrogens 0.625 mg and 0.45 mg/BZA 20 mg	2 years	1583 and 1583 postmenopausal women respectively	Conjugated estrogens/BZA provides endometrial protection without increasing breast pain/density, vaginal bleeding, or ovarian cysts	[75]
Estrogen and ER modulator	BZA 20 mg/CE 0.45 or 0.625 mg, BZA 20 mg.	12 weeks	Postmenopausal, non-hysterectomized women (*n* = 652) with symptoms of moderate to severe vulvar/vaginal atrophy	Shown to significantly improve sexual function and quality-of-life measures in symptomatic postmenopausal women	[142]
Estradiol	Vaginal 4 μg and 10 μg estradiol (E2)	12 weeks	25 eligible women	Because of endometrial progesterone receptor expression, vaginal E2 would not be expected to stimulate endometrial hyperplasia leading to moderate to severe dyspareunia which is a symptom of vulvar and vaginal atrophy and cancer	[143]
Selective serotonin-reuptake inhibitors (SSRIs)	7.5 mg oral paroxetine or placebo daily	16 weeks	80 women	Paroxetine significantly reduced hot flashes in weekly frequency and severity in gynecological cancer survivors	[144]
